# Shotgun metagenomic sequencing of swab samples from Japanese university campuses

**DOI:** 10.1128/mra.00210-24

**Published:** 2024-06-05

**Authors:** Megumu Tsujimoto, Dewa A. P. Rasmika Dewi, Christopher E. Mason, Yuh Shiwa, Haruo Suzuki

**Affiliations:** 1 Faculty of Environment and Information Studies, Keio University, Fujisawa, Kanagawa, Japan; 2 School of Public Health and Preventive Medicine, Monash University, Melbourne, Australia; 3 School of Medicine and Health Sciences, Udayana University, Bali, Indonesia; 4 Department of Physiology and Biophysics, Weill Cornell Medicine, New York, New York, USA; 5 The HRH Prince Alwaleed Bin Talal Bin Abdulaziz Alsaud Institute for Computational Biomedicine, Weill Cornell Medicine, New York, New York, USA; 6 The WorldQuant Initiative for Quantitative Prediction, Weill Cornell Medicine, New York, New York, USA; 7 Department of Molecular Microbiology, Tokyo University of Agriculture, Tokyo, Japan; 8 NODAI Genome Research Center, Tokyo University of Agriculture, Tokyo, Japan; 9 Institute for Advanced Biosciences, Keio University, Tsuruoka, Yamagata, Japan; Montana State University, Bozeman, Montana, USA

**Keywords:** shotgun metagenome sequences, Japanese university campuses, DNA Data Bank of Japan, swab samples, air samples (negative control), surface samples (table, seat, floor), microbial community, microbiology of the built environment, urban microbiome, MetaSUB international consortium

## Abstract

We obtained shotgun metagenome sequences from swab samples obtained through 3-minute swabbing of different surfaces and the air within buildings at three university campuses in part of the Greater Tokyo Area in Japan. These data aid in understanding built environment microbial communities and elucidate various microbial profiles across different locations.

## ANNOUNCEMENT

Human life mostly occurs indoors, within the confines of homes, schools, and workplaces. Interactions between human commensal microorganisms and indoor microbiomes in built environments (1) have implications for human health and potential disease transmission (2). University campuses, characterized by high human density and diverse room/building usage, provide distinct opportunities for investigating microbial communities within built environments (3, 4).

**Fig 1:**
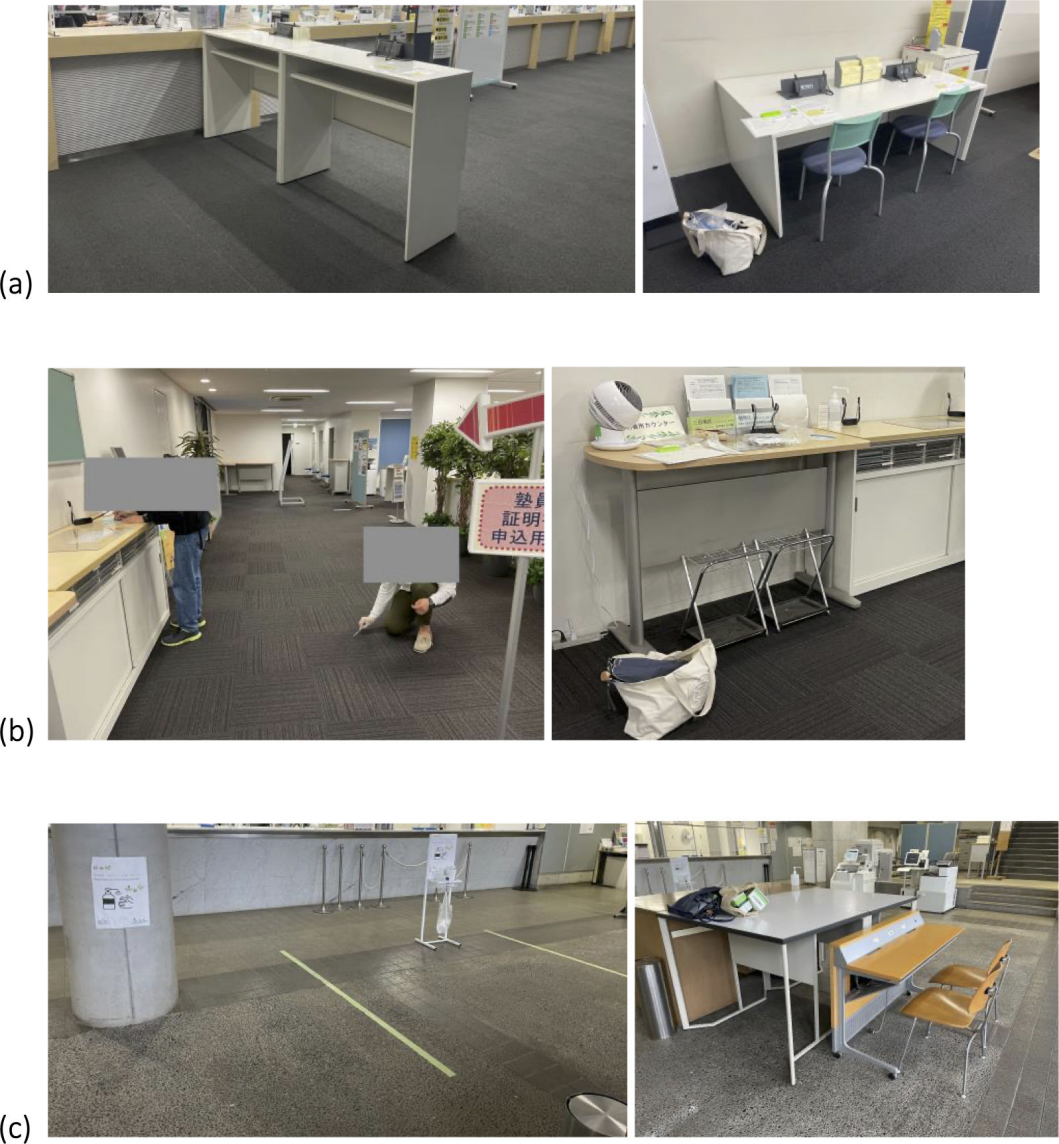
Sample collection sites in Keio University. (a) Hiyoshi Campus, (b) Mita Campus, and (c) Shonan Fujisawa Campus.

The present study focused on three specific campuses in Keio University—Mita, Hiyoshi, and Shonan Fujisawa—to determine the variations in microbial communities based on location. Mita, located in Minato City in Tokyo’s metropolitan area, is the university’s main campus. Hiyoshi is located in Yokohama, part of Kanagawa Prefecture’s urban landscape, whereas the Shonan Fujisawa Campus is located in Fujisawa, a rural suburban area in Kanagawa Prefecture. Interactions with human-associated microbial sources (oral, fecal, vaginal, etc.) vary depending on surfaces within built environments (seats, floors, etc.). In September 2021, during the fifth wave of the COVID-19 pandemic in Japan, we collected swab samples from three types of surfaces—tables, seats, and floors—within buildings at Mita, Hiyoshi, and Shonan Fujisawa Campuses (Fig. 1). Specifically, samples were obtained from the Office of Student Services at Mita and Hiyoshi Campuses and from the main building (Alpha Building) at Shonan Fujisawa Campus. These service points are frequented by members affiliated with the university, including students, staff, and faculty.

We employed sampling and sample processing procedures as detailed in a previous report (5). Briefly, swab samples were collected using Isohelix swabs (Cell Projects Ltd., Maidstone, UK) and tubes containing 400 μL of DNA/RNA Shield medium (Zymo Research Co., CA, USA) provided by the MetaSUB International Consortium (http://metasub.org). To estimate potential contamination introduced during several operational steps, including sample collection and processing (6), we used air samples as “negative control,” designated as “background control” (7), with swabs exposed to the air for 3 minutes, as demonstrated by Danko et al. (7). Metagenomic DNA extraction was done using the ZymoBiomics DNA Miniprep Kit (Zymo Research Co.) according to the manufacturer’s instructions and quantified using Qubit double-stranded DNA high-sensitivity assay kits (ThermoFisher Scientific Inc., MA, USA) according to the manufacturer’s procedure. Shotgun metagenomic sequencing was performed using the 2 × 150-bp paired-end read DNBSEQ-G400RS high-throughput sequencing set (MGITech Co., Tokyo, Japan) performed at Genome Lead Co. Ltd. (Kagawa, Japan), and the metagenomic library was prepared using the MGIEasy FS DNA Library Prep Set (MGITech Co.) with 10 cycles of real-time PCR amplification.

We present shotgun metagenomic sequences obtained from 12 swab samples collected on university campuses. The air samples (negative control) exhibited fewer raw sequencing reads (num_seqs in Table 1), ranging from 68,434 to 264,890, than the surface (table, seat, and floor) samples, which ranged from 1,895,779 to 12,814,699 (Table 1). We intend to perform a comprehensive comparative analysis of shotgun metagenomic sequences from other urban built environments, such as university campuses and subway stations, in different cities and publish the findings in a separate future publication.

**TABLE 1:** Shotgun metagenomic sequence data for 12 swab samples

Sample_name	lat_lon^*a*^	Location^*b*^	Sample_type/place^*c*^	BioSample^*d*^	Run^*e*^	num_seqs^*f*^
DNBSEQ_S02	35.552 N 139.647 E	Hiyoshi	Negative Control	SAMD00520046	DRR413340	98565
DNBSEQ_S31	35.552 N 139.647 E	Hiyoshi	Table	SAMD00520047	DRR413341	3952797
DNBSEQ_S32	35.552 N 139.647 E	Hiyoshi	Seat	SAMD00520048	DRR413342	3370231
DNBSEQ_S33	35.552 N 139.647 E	Hiyoshi	Floor	SAMD00520049	DRR413343	3258126
DNBSEQ_S34	35.388 N 139.428 E	Shonan Fujisawa	Negative Control	SAMD00520050	DRR413344	68434
DNBSEQ_S35	35.388 N 139.428 E	Shonan Fujisawa	Table	SAMD00520051	DRR413345	3735202
DNBSEQ_S36	35.388 N 139.428 E	Shonan Fujisawa	Seat	SAMD00520052	DRR413346	4017081
DNBSEQ_S37	35.388 N 139.428 E	Shonan Fujisawa	Floor	SAMD00520053	DRR413347	12814699
DNBSEQ_S38	35.648 N 139.743 E	Mita	Negative Control	SAMD00520054	DRR413348	264890
DNBSEQ_S39	35.648 N 139.743 E	Mita	Table	SAMD00520055	DRR413349	1895779
DNBSEQ_S40	35.648 N 139.743 E	Mita	Seat	SAMD00520056	DRR413350	2424992
DNBSEQ_S41	35.648 N 139.743 E	Mita	Floor	SAMD00520057	DRR413351	4161440

^*a*^ lat_lon, Geographical coordinates (latitude and longitude) indicating sample collection locations. (https://www.ddbj.nig.ac.jp/biosample/attribute-e.html).

^*b*^ Location, Keio University campuses: Hiyoshi, Mita, and Shonan Fujisawa.

^*c*^ Sample_type/place, Negative Control, Table, Seat, and Floor.

^*d*^ BioSample, DDBJ BioSample accession number.

^*e*^ Run, DDBJ Sequence Read Archive (DRA) accession number.

^*f*^ num_seqs, Number of raw sequencing reads.

## Data Availability

The project information is available under the DNA Data Bank of Japan (DDBJ) BioProject accession number PRJDB14136 and the umbrella BioProject accession number PRJDB13760. Raw sequencing reads were deposited in the DDBJ Sequence Read Archive (DRA). Table 1 lists DDBJ BioSample accession numbers (in the "BioSample" column) and DDBJ Sequence Read Archive (DRA) accession numbers (in the "Run" column).
